# Weight management with orlistat in type 2 diabetes: an electronic health records study

**DOI:** 10.3399/BJGP.2023.0684

**Published:** 2024-09-17

**Authors:** Shraboni Ghosal, Neil Heron, Kayleigh J Mason, James Bailey, Kelvin P Jordan

**Affiliations:** School of Medicine, Keele University, Keele; research associate (postdoctoral), School of Health Sciences, University of Manchester, Manchester.; School of Medicine, Keele University, Keele; clinical lecturer, Centre for Public Health, Queen’s University Belfast, Belfast.; School of Medicine, Keele University, Keele.; School of Medicine, Keele University, Keele.; School of Medicine, Keele University, Keele.

**Keywords:** body mass index, obesity, prediabetes, primary care, type 2 diabetes, weight loss

## Abstract

**Background:**

Orlistat is recommended as an adjunct to diet and exercise for weight loss in the treatment of type 2 diabetes mellitus (T2DM).

**Aim:**

To explore associations between patient characteristics and orlistat prescribing, and to determine associations of orlistat with weight loss in T2DM and prediabetes.

**Design and setting:**

Cohort study using anonymised health records from a UK database of general practice.

**Method:**

The UK Clinical Practice Research Datalink (CPRD) Aurum database was searched to compile a cohort of patients aged ≥18 years, first diagnosed with T2DM or prediabetes in 2016 or 2017. Once the data had been collated, multivariable logistic regression models were used to determine associations with starting orlistat and stopping it early (<12 weeks of prescriptions) and orlistat’s associations with weight loss in those who had not been prescribed second-line antidiabetic medications.

**Results:**

Out of 100 552 patients with incident T2DM or prediabetes, 655 (0.8%) patients with T2DM and 128 (0.7%) patients with prediabetes were prescribed orlistat. Younger people, females, those in areas of deprivation, current smokers, those coprescribed metformin, and those recorded as having hypertension were statistically significantly more likely to be prescribed orlistat; higher baseline glycated haemoglobin levels were associated with early stopping. In comparison with patients not on orlistat, those who continued using it for ≥12 weeks were more likely to lose ≥5% weight (adjusted odds ratio [AOR] 1.69, 95% confidence interval [CI] = 1.07 to 2.67) but those who stopped orlistat early were less likely to lose ≥5% weight (AOR 0.56, 95% CI = 0.29 to 1.09).

**Conclusion:**

Orlistat was significantly associated with weight loss in patients with T2DM and prediabetes when taken for at least 12 weeks; however, it was infrequently prescribed and often taken for <12 weeks. Orlistat may be a useful adjunct to lifestyle modifications for patients with T2DM and prediabetes, but barriers to continued use means it may not be effective for everyone in managing weight loss.

## Introduction

Type 2 diabetes mellitus (T2DM) is a major public health burden and remains a health challenge globally.^1^ In the UK, diabetes diagnoses have more than doubled in the past 15 years to >5 million people having a diabetes diagnosis in 2024.^2^ Prediabetes prevalence has more than tripled, with 30% of adults — or one in nine — in England having prediabetes in 2024.^3^ Diabetes-related complications — such as blindness, cardiovascular disease, nerve damage, or kidney disease — are a major cause of disability, reduced quality of life, or even death.^4^ Obesity increases the risk of T2DM^5^^,^^6^ and its complications.^7^^,^^8^ In the UK, 90% of those with T2DM are obese or overweight; however, obesity is modifiable.^9^ Weight loss is an important therapeutic strategy in those who are overweight or obese and have T2DM or prediabetes.^9^^,^^10^ Although lifestyle modifications are recommended,^10^ weight loss and management can be challenging for many,^11^ so additional interventions, such as pharmacotherapy, may be necessary.

Orlistat (tetrahydrolipstatin) is an oral treatment that has been approved by the National Institute for Health and Care Excellence (NICE) to treat obesity in the UK; it has a licence to be prescribed as an adjunct to diet and exercise for those with a body mass index (BMI) ≥30 kg/m^2^ or a BMI ≥28 kg/m^2^ with risk factors, such as T2DM.^12^^–^^14^ Orlistat (as with liraglutide and semaglutide) is available through the UK’s NHS for obesity treatment.^12^^–^^15^ Liraglutide and semaglutide are injectable and can be expensive, whereas orlistat is an oral medication with evidence of cost-effectiveness.^16^ Orlistat is known to cause side-effects, such as flatulence, diarrhoea, gastrointestinal disorders, abdominal pain, fatty stools, or faecal incontinence;^15^^,^^17^ use of it can be stopped early due to this or if ≤5% weight is lost within 12 weeks.^17^ Although randomised controlled trials (RCTs) indicate that orlistat is effective in T2DM treatment,^18^^–^^20^ real-world evidence on the extent to which it is prescribed and continued for >12 weeks, and its impact in patients who are overweight/obese and have T2DM or prediabetes, is limited.

The objectives of this study were to determine:

- patient characteristics associated with orlistat prescribing and with stopping orlistat early in T2DM in UK primary care (objective one); and
- orlistat’s association with weight loss and change in other key diabetes markers — for example, glycaemic parameters and blood pressure (BP) — in patients with T2DM or prediabetes (objective two).

**Table:** How this fits in

Previously published randomised controlled trials have shown the effectiveness of orlistat as an adjunct to lifestyle/behavioural factors for weight loss in patients with T2DM and prediabetes. Orlistat can be prescribed for weight loss for these patients, but evidence on the extent of its use and association with weight loss in real-world settings are lacking. The study presented here showed that orlistat used for ≥12 weeks is associated with ≥5% weight loss in patients with T2DM and prediabetes; however, it is infrequently used in T2DM and often stopped early. There appear to be barriers to continuing orlistat long-term in patients with T2DM and prediabetes, and it may not be effective for everyone.

## Method

### Study design and setting

The study used the UK Clinical Practice Research Datalink (CPRD) Aurum database,^21^^,^^22^ with the linked neighbourhood-level Index of Multiple Deprivation (IMD) 2019^23^ used as a measure of socioeconomic status. CPRD Aurum contains routinely collected anonymised data from primary care general practices in the UK (currently about 13 million patients are registered, covering 20% of the UK population), and predominantly represents the broader English population.^24^

### Study population

The study population comprised patients aged ≥18 years, who had a first diagnosis of T2DM or prediabetes in 2016 or 2017. The index date was the date of the first T2DM or prediabetes diagnosis. Patients also had to have at least 2 years’ records in the CPRD prior to diagnosis and at least 2 years’ follow-up data. Read codes were used in UK primary care prior to 2018 to record diagnoses, and code lists for T2DM and prediabetes were finalised in consensus with the other authors of this article (see Supplementary Table S1).

For objective two, the authors included only those patients with a baseline BMI ≥28 kg/m^2^ and excluded those with records of second-line antidiabetic medications/insulin, as these may also result in weight change.^25^^,^^26^ Supplementary Figure S1 outlines the derivation of the study population in more detail.

### Exposure

The exposure for objective two was orlistat prescription at time of diagnosis (within 12 weeks before, or 12 weeks after, diagnosis). Three groups were compared:

- prescribed orlistat within ±12 weeks of diagnosis and stopped early (group 1);
- prescribed orlistat within ±12 weeks of diagnosis date and continued past 12 weeks (group 2); and
- no orlistat within ±12 weeks of diagnosis (group 3).

Continued use was defined as having at least one orlistat prescription >12 weeks after the initial prescription, with a gap of <6 months since the previous prescription.

### Outcomes

#### Objective one

The outcome was an orlistat prescription from 12 weeks before up until 2 years after diagnosis. The 12 weeks before diagnosis was included in case GPs had prescribed orlistat during the diagnosis window for diabetes. ‘Stopping orlistat early’ was defined as no recorded orlistat prescription after 12 weeks of the initial prescription.^13^ This implies that if there was no recorded orlistat prescription after 12 weeks of the initial prescription, it was assumed that orlistat was stopped as per NICE guidelines.^13^ NICE guidelines recommend that orlistat therapy should be continued beyond 12 weeks only if the person has lost at least 5% of their initial body weight since starting treatment.^13^

#### Objective two

The primary outcome was weight loss of ≥5% from baseline to last recorded weight:

- prior to 12 months after diagnosis; and
- prior to 24 months after diagnosis.

Those without weight recordings at baseline or follow-up were excluded.

For secondary outcomes, association of orlistat with actual change in weight, glycated haemoglobin (HbA1c) levels, and systolic BP were assessed using the last recorded measurement within 12 months and 24 months of diagnosis.

### Covariates

The covariates at baseline were selected by consensus of the research team as being potentially associated with the prescribing of orlistat. These were age (at T2DM/prediabetes diagnosis), gender, hypertension, HbA1c levels, metformin prescription, geographical region, IMD quintile, smoking status, and baseline BMI recordings (that is, the last measurement at baseline). Baseline BMI, HbA1c level, hypertension status, and smoking status were defined based on the last recordings/status in the 2 years prior to diagnosis or at diagnosis. Metformin prescription was based on a record of 12 weeks either side of diagnosis. Patients without a recorded baseline BMI were included alongside those with a baseline BMI ≤25 kg/m^2^. Geographical regions were combined and categorised into: North (North East, North West, and Yorkshire and the Humber); Midlands and East (East and West Midlands and East of England); and South and London (South East, South West, and London).

### Patient and public involvement

A patient and public involvement and engagement session involving people with T2DM/prediabetes/carers was conducted to inform and interpret the findings, and to ensure that patient and public perspectives were taken into consideration.

### Sensitivity analysis

To assess the impact of missing data, patients with no recorded values (for example, weight/BMI) during follow-up were included by coding them as missing categories. A number of sensitivity analyses were also undertaken to assess associations with orlistat prescribing where those with a BMI ≤25 kg/m^2^ had been excluded.

### Statistical methods

#### Objective one

Associations of covariates with orlistat prescription in patients with T2DM were determined using logistic regression and quantified by adjusted odds ratios (AORs) (adjusting for all covariates) with 95% confidence intervals (CIs). Analysis was repeated for prescribing at time of diagnosis (±12 weeks of diagnosis date). For those with an orlistat prescription, associations with stopping orlistat early were also determined using logistic regression. The analysis was repeated excluding those with a BMI ≤25 kg/m^2^.

#### Objective two

Associations between orlistat prescribing and change in weight were modelled using:

- logistic regression for weight loss, ≥5% versus <5%; and
- linear regression for actual change in weight, adjusting for all covariates and time between baseline and follow-up BMI measurement.

Analyses were performed separately for 1 and 2 years after diagnosis. Patients with no recorded outcome values (for example, weight/BMI) during follow-up were excluded (complete case analysis). In model 1, orlistat (unadjusted) was used as the explanatory variable on its own, with weight change as the outcome. For model 2, baseline BMI was added. Model 3 was adjusted for all covariates. For model 4 (not shown), interaction of orlistat with metformin (multiplicative) was added to ascertain whether metformin moderated orlistat’s association with outcomes. The analysis was repeated for secondary outcomes of change in HbA1c levels and systolic BP.

## Results

### Objective one

In total, 100 552 patients (82 696 with T2DM and 17 856 with prediabetes) were included in the study. Patient characteristics are shown in Table 1. There were a total of 783 (0.8%) patients with T2DM or prediabetes who were prescribed orlistat (655 [0.8%] patients with T2DM and 128 [0.7%] patients with prediabetes) (Table 1); of these, 42% received only a single orlistat prescription (data not shown). Patients with T2DM received a total of 2264 orlistat prescriptions at a rate of 173 per 100 person–years during the study period, and patients with prediabetes received a total of 429 orlistat prescriptions, at a rate of 168 per 100 person–years (approximately one or two orlistat prescriptions per person per year in those with at least one prescription).

**Table 1.** Baseline characteristics of study population

**Baseline characteristic**	**Patients with T2DM or prediabetes, *n* = 100 552**	**Patients with T2DM, *n* = 82 696**	**Patients with prediabetes, *n* = 17 856**
**Age, years, mean (SD)**	62.5 (13.9)	62.1 (13.8)	64.0 (13.9)

**Gender, *n* (%)**			
Male	55 028 (54.7)	46 468 (56.2)	8560 (47.9)
Female	45 524 (45.3)	36 228 (43.8)	9296 (52.1)

**Body mass index, *n* (%)**			
Baseline measurement	72 894 (72.5)	61 519 (74.4)	11 375 (63.7)
<18.5 kg/m^2^: underweight	470 (0.6)	305 (0.5)	165 (1.5)
18.5–24.9 kg/m^2^: normal	9443 (13.0)	7300 (11.9)	2143 (18.8)
25–29.9 kg/m^2^: overweight	24 111 (33.1)	19 756 (32.1)	4355 (38.3)
≥30 kg/m^2^: obese	38 870 (53.3)	34 158 (55.5)	4712 (41.4)
Mean kg/m^2^ (SD)	31.3 (6.5)	31.6 (6.6)	29.6 (5.8)

**IMD quintile, *n* (%)**			
1 (least deprivation)	18 762 (18.7)	15 140 (18.3)	3622 (20.3)
2	18 311 (18.2)	15 084 (18.2)	3227 (18.1)
3	18 901 (18.8)	15 448 (18.7)	3453 (19.3)
4	21 883 (21.8)	18 025 (21.8)	3858 (21.6)
5 (most deprivation)	21 412 (21.3)	17 956 (21.7)	3456 (19.4)
Missing	1283	1043	240

**Region, *n* (%)**			
1: North of England	22 043 (21.9)	18 292 (22.1)	3751 (21.0)
2: Midlands and East of England	27 632 (27.5)	22 974 (27.8)	4658 (26.1)
3: South of England and London	49 465 (49.2)	40 442 (48.9)	9023 (50.5)
Missing	1412	988	424

**Baseline smoking status, *n* (%)**	77 151 (76.7)	64 318 (77.8)	12 833 (71.9)
Ex-smoker	19 015 (24.6)	15 928 (24.8)	3087 (24.1)
Current smoker	21 842 (28.3)	18 366 (28.6)	3476 (27.1)
Non-smoker	36 294 (47.0)	30 024 (46.7)	6270 (48.9)

**Prescribed orlistat, *n* (%)**	783 (0.8)	655 (0.8)	128 (0.7)

*IMD = Index of Multiple Deprivation. SD = standard deviation. T2DM = type 2 diabetes mellitus.*

Details of the associations between baseline characteristics and orlistat prescriptions are shown in Table 2. Of patients with T2DM, those with a BMI ≥30 kg/m^2^ were more likely to be prescribed orlistat, as were females and younger patients. Those from the North of England were more likely to be prescribed orlistat than those from the South/London. Orlistat was also associated with metformin prescriptions, current smoking, and hypertension. Patients from more-affluent areas were less likely to be prescribed orlistat. Higher baseline HbA1c values decreased the probability of orlistat prescribing. Associations were similar with prescriptions within ±12 weeks of diagnosis (Table 2) and after excluding those with BMI ≤25 kg/m^2^ (see Supplementary Table S2).

**Table 2.** Associations of baseline characteristics with orlistat prescriptions in patients with T2DM, *N* = 82 696

**Independent variables**		**Orlistat prescription at any time**			**Prescription within ±12 weeks of diagnosis**		

**Independent variable**	**Total, *n***	**Orlistat, *n***	**OR (95% CI)**	**AOR (95% CI)**	**Orlistat, *n***	**OR (95% CI)**	**AOR (95% CI)**
**Age, years**							
18–39.9	4709	117	2.34 (1.90 to 2.87)	2.51 (2.01 to 3.12)	40	2.19 (1.55 to 3.10)	2.30 (1.60 to 3.31)
≥65	35 828	83	0.21 (0.17 to 0.27)	0.26 (0.20 to 0.34)	34	0.24 (0.17 to 0.35)	0.32 (0.22 to 0.47)
40–64.9(reference)	42 159	455	1	1	164	1	1

**Gender**							
Female	36 228	391	1.91 (1.63 to 2.23)	2.02 (1.71 to 2.39)	139	1.80 (1.39 to 2.34)	1.82 (1.39 to 2.39)
Male (reference)	46 468	264	1	1	99	1	1

**Body mass index**							
18.5–24.9kg/m^2^ or not recorded	28 782	108	0.25 (0.20 to 0.30)	0.27 (0.21 to 0.33)	29	0.17 (0.12 to 0.26)	0.18 (0.12 to 0.27)
25–29.9 kg/m^2^	19 756	30	0.10 (0.07 to 0.14)	0.14 (0.09 to 0.20)	11	0.10 (0.05 to 0.18)	0.12 (0.06 to 0.23)
≥30 kg/m^2^ (reference)	34 158	517	1	1	198	1	1

**Region^a^**							
1: North of England	18 292	185	1.30 (1.08 to 1.56)	1.25 (1.03 to 1.51)	68	1.29 (0.95 to 1.74)	1.24 (0.90 to 1.70)
2: Midlands and East of England	22 974	148	0.82 (0.68 to 1.00)	0.96 (0.78 to 1.17)	51	0.77 (0.55 to 1.07)	0.87 (0.62 to 1.22)
3: South of England and London (reference)	40 442	316	1	1	117	1	1

**Index of Multiple Deprivation quintile^b^**							
1 (least deprived)	15 140	63	0.37 (0.28 to 0.49)	0.68 (0.50 to 0.91)	20	0.35 (0.21 to 0.58)	0.67 (0.40 to 1.11)
2	15 084	107	0.63 (0.50 to 0.80)	0.93 (0.73 to 1.20)	43	0.76 (0.52 to 1.12)	1.13 (0.75 to 1.70)
3	15 448	104	0.60 (0.47 to 0.76)	0.82 (0.64 to 1.05)	42	0.73 (0.50 to 1.07)	1.05 (0.70 to 1.56)
4	18 025	170	0.84 (0.68 to 1.03)	1.02 (0.82 to 1.26)	62	0.92 (0.65 to 1.30)	1.12 (0.78 to 1.61)
5 (most deprivation) (reference)	17 956	202	1	1	67	1	1

**Blood glucose**							
Baseline HbA1c level recorded	77 109	631	1.00 (1.00 to 1.01)	0.990 (0.99 to 1.00)	231	0.99 (0.99 to 1.00)	0.98 (0.97 to 0.99)
Metformin	35 963	246	2.17 (1.86 to 2.55)	1.87 (1.56 to 2.24)	143	1.96 (1.51 to 2.54)	1.83 (1.37 to 2.46)
No metformin (reference)	46 733	409	1	1	95	1	1

**Smoking status**							
Not recorded	18 378	144	1.09 (0.88 to 1.34)	1.41 (1.12 to 1.78)	56	1.17 (0.83 to 1.66)	1.83 (1.27 to 2.63)
Ex-smoker	15 928	112	0.97 (0.77 to 1.22)	1.26 (0.99 to 1.61)	34	0.82 (0.55 to 1.23)	1.01 (0.65 to 1.55)
Current smoker	18 366	182	1.38 (1.13 to 1.68)	1.29 (1.05 to 1.59)	70	1.47 (1.06 to 2.03)	1.47 (1.05 to 2.06)
Non-smoker (reference)	30 024	217	1	1	78	1	1

**Hypertension**							
Recorded hypertension	27 947	240	1.13 (0.97 to 1.33)	1.27 (1.07 to 1.51)	81	1.01 (0.77 to 1.32)	1.09 (0.82 to 1.44)
Nohypertension (reference)	54 749	415	1	1	157	1	1

*AOR = adjusted odds ratio. HbA1c = glycated haemoglobin. OR = odds ratio. T2DM = type 2 diabetes mellitus.*

a *Data for 988 patients are missing.*

b *Data for 1043 patients are missing.*

Of 655 patients with T2DM who had orlistat prescriptions, 292 (44.6%) had stopped early. Higher baseline HbA1c levels were significantly associated with early stopping (AOR 1.01/unit increase, 95% CI = 1.00 to 1.02). After adjustment, no other statistically significant associations with early stopping of orlistat were found (Table 3).

**Table 3.** Associations of baseline characteristics with stopping orlistat prescription early in patients with T2DM, *N* = 655

**Independent variable**	**Patients with T2DM, *n***	**OR (95% CI)**	**AOR (95% CI)**
**Age, years**			
18–39.9	117	0.66 (0.44 to 0.99)	0.70 (0.45 to 1.10)
≥65	83	0.97 (0.61 to 1.56)	1.06 (0.64 to 1.75)
40–64.9 (reference)	455	1	1

**Gender**			
Female	264	1.19 (0.87 to 1.63)	1.09 (0.77 to 1.53)
Male (reference)	391	1	1

**Body mass index**			
18.5–24.9 kg/m^2^ or not recorded	108	0.85 (0.56 to 1.29)	0.79 (0.49 to 1.28)
25–29.9 kg/m^2^	30	1.18 (0.56 to 2.51)	1.40 (0.63 to 3.14)
≥30 kg/m^2^ (reference)	517	1	1

**Region^a^**			
1: North of England	185	1.47 (1.020 to 2.13)	1.37 (0.91 to 2.05)
2: Midlands and East of England	148	1.20 (0.81 to 1.78)	1.18 (0.78 to 1.79)
3: South of England and London (reference)	316	1	1

**Index of Multiple Deprivation quintile^b^**			
1 (least deprivation)	63	0.62 (0.35 to 1.09)	0.68 (0.38 to 1.24)
2	107	1.08 (0.67 to 1.74)	1.14 (0.68 to 1.90)
3	104	1.12 (0.69 to 1.81)	1.18 (0.70 to 1.98)
4	170	0.73 (0.48 to 1.10)	0.80 (0.52 to 1.24)
5 (most deprivation) (reference)	202	1	1

**Blood glucose**			
Baseline HbA1c level recorded	631	1.01 (1.00 to 1.02)	1.01 (1.00 to 1.02)
Metformin	246	0.87 (0.63 to 1.19)	0.92 (0.63 to 1.33)
No metformin (reference)	409	1	1

**Smoking status**			
Not recorded	144	0.82 (0.54 to 1.26)	0.87 (0.54 to 1.39)
Ex-smoker	112	0.91 (0.58 to 1.45)	0.77 (0.47 to 1.28)
Current smoker	182	0.90 (0.60 to 1.34)	0.91 (0.60 to 1.40)
Non-smoker (reference)	217	1	1

**Hypertension**			
Recorded hypertension	240	1.38 (1.00 to 1.90)	1.36 (0.95 to 1.93)
No hypertension (reference)	415	1	1

*AOR = adjusted odds ratio. HbA1c = glycated haemoglobin. OR = odds ratio. T2DM = type 2 diabetes mellitus.*

a *Data for 6 patients missing.*

b *Data for 9 patients missing.*

### Objective two

In total, 28 526 patients with T2DM and prediabetes for the outcomes of weight loss after 1 year, and 33 873 patients with T2DM and prediabetes after 2 years, were included in the study (see Supplementary Figure S1). At 1 year and at 2 years, patients in all orlistat groups (stopped orlistat early, continued orlistat ≥12 weeks, and no orlistat within 12 weeks of diagnosis) had lost some weight (Table 4). Those who had continued orlistat for ≥12 weeks had lost the most weight (mean 4.17 kg [standard deviation {SD} 6.09]) at 2 years, whereas those that had stopped early had lost the least (1.89 kg [SD 9.12]) (see Supplementary Table S3).

**Table 4.** Associations of orlistat prescription with weight loss ≥5% and with actual weight loss in patients with T2DM and prediabetes

**Group**	***n***	**Lost ≥5% weight, *n* (%)**	**Logistic regression models 1–3**		

			**Model 1: unadjusted, OR (95% CI)**	**Model 2: adjusted for baseline BMI, AOR (95% CI)**	**Model 3: adjusted for all covariates,^a^ AOR (95% CI)**
**1 year, *N* = 28 526**					
Group 1 — stopped early	61	12 (19.7)	0.55 (0.29 to 1.03)	0.50 (0.27 to 0.95)	0.56 (0.29 to 1.09)
Group 2 — continued	80	34 (42.5)	1.66 (1.06 to 2.59)	1.53 (0.98 to 2.39)	1.69 (1.07 to 2.67)
Group 3 — no orlistat (reference)	28 385	8752 (30.8)	1	1	1

**2 years, *N* = 33 873**					
Group 1 — stopped early	69	17 (24.6)	0.67 (0.39 to 1.16)	0.60 (0.4 to 1.04)	0.68 (0.39 to 1.18)
Group 2 — continued	83	37 (44.6)	1.66 (1.07 to 2.55)	1.49 (0.97 to 2.30)	1.55 (0.99 to 2.43)
Group 3 — no orlistat (reference)	33 721	11 026 (32.7)	1	1	1

**Group**	***n***	**Linear regression models 1–3**		

		**Model 1: unadjusted, β-estimate (95% CI)**	**Model 2: adjusted for baseline BMI, β-estimate (95% CI)**	**Model 3: adjusted for all covariates,^a^ β-estimate (95% CI)**
**1 year, *N* = 28 526**				
Group 1 — stopped early	61	−1.32 (−2.88 to 0.24)	−2.29 (−3.84 to −0.74)	−1.79 (−3.35 to −0.24)
Group 2 — continued	80	1.34 (−0.03 to 2.70)	0.41 (−0.95 to 1.76)	0.64 (−0.71 to 1.10)
Group 3 — no orlistat (reference)	28 385	1	1	1

**2 years, *N* = 33 873**				
Group 1 — stopped early	69	−1.19 (−2.84 to 0.47)	−2.42 (−4.06 to −0.78)	−2.03 (−3.68 to −0.38)
Group 2 — continued	83	1.10 (−0.41 to 2.62)	−0.09 (−1.58 to 1.41)	0.11 (−1.40 to 1.62)
Group 3 — no orlistat (reference)	33 721	1	1	1

a *Covariates included: age (18–39.9 years, 40–64.9 years, or ≥65 years); gender (male or female); baseline body mass index; region (1: North of England, 2: Midlands and East of England, or 3: South of England and London); Index of Multiple Deprivation quintiles 1–5; baseline HbA1c; metformin prescription (metformin or no metformin); smoking status (not recorded, ex-smoker, current smoker, or non-smoker); hypertension (recorded hypertension or no hypertension).*

*AOR = adjusted odds ratio. BMI = body mass index. OR = odds ratio. T2DM = type 2 diabetes mellitus.*

At 1 year, 8798 (30.8%) had ≥5% weight loss (mean baseline weight 97.73 kg [SD 19.36] versus 1 year 88.23 kg [SD 17.83]); this comprised those who had continued orlistat (42.5%), stopped orlistat early (19.7%), and had no orlistat (30.8%). After 2 years, 11 080 (32.7%) patients had lost ≥5% weight (mean baseline weight 97.31 kg [SD 19.55] versus 2 years 87.33 kg [SD 17.87]); this comprised those who had continued orlistat (44.6%), stopped orlistat early (24.6%), and had no orlistat (32.7%) (Table 4). In unadjusted analyses, those who continued orlistat were more likely to lose ≥5% weight in 1 year (versus no orlistat OR 1.66, 95% CI = 1.06 to 2.59), whereas those who stopped early were less likely to do so, although this was not statistically significant (OR 0.55, 95% CI = 0.29 to 1.03). A weight loss of 5% was equivalent to losing ∼5 kg, on average, in this population. After adjustment for all covariates, the strength of these associations was maintained (continued versus no orlistat OR 1.69, 95% CI = 1.07 to 2.67; stopped early versus no orlistat OR 0.56, 95% CI = 0.29 to 1.09). Similar associations were found after 2 years (continued versus no orlistat OR 1.55, 95% CI = 0.99 to 2.43; stopped early versus no orlistat OR 0.68, 95% CI = 0.39 to 1.18) (Table 4). There was no significant interaction of metformin and orlistat with weight loss in 1 or 2 years (data not shown).

When analysing actual weight loss, those who stopped orlistat early lost significantly less weight (versus no orlistat adjusted β-estimate = −1.79 kg, 95% CI = −3.35 to −0.24) and those who continued lost more weight but this was not statistically significant (versus no orlistat adjusted β-estimate = 0.64 kg, 95% CI = −0.71 to 1.10) over 1 year; findings were similar after 2 years (Table 4).

Those who stopped orlistat early had significantly smaller reductions in HbA1c after 1 year (versus no orlistat adjusted β-estimate = −5.08 mmol/mol, 95% CI = −8.45 to −1.72) and after 2 years (versus no orlistat adjusted β-estimate = −4.40 mmol/mol, 95% CI = −7.21 to −1.59) (Table 5). There was no difference in the change in HbA1c level between those who continued orlistat and those not prescribed it (data not shown). Those who continued orlistat had significantly greater reductions in systolic BP after 1 year (versus no orlistat adjusted β-estimate = 3.98, 95% CI = 0.44 to 7.52) (Table 5).

**Table 5.** Associations of orlistat with changes in HbA1c level and blood pressure in patients with T2DM and prediabetes (fully adjusted)

**Groups 1–3**	**HbA1c level, adjusted β-estimate (95% CI)**	**Systolic blood pressure, adjusted β-estimate (95% CI)**
**1 year, *n***	27 995	31 140
Group 1 — stopped early	−5.08 (−8.45 to −1.72)	2.71 (−1.34 to 6.76)
Group 2 — continued	1.55 (−1.38 to 4.48)	3.98 (0.44 to 7.52)
Group 3 — no orlistat (reference)	1	1

**2 years, *n***	36 921	38 657
Group 1 — stopped early	−4.40 (−7.21 to −1.59)	−0.77 (−4.27 to 2.72)
Group 2 — continued	−0.51 (−3.20 to 2.19)	2.61 (−0.72 to 5.93)
Group 3 — no orlistat (reference)	1	1

a *Covariates included: age (18–39.9 years, 40–64.9 years, or ≥65 years); gender (male or female); baseline body mass index; region (1: North of England, 2: Midlands and East of England, or 3: South of England and London); Index of Multiple Deprivation quintiles 1–5; baseline HbA1c; metformin prescription (metformin or no metformin); smoking status (not recorded, ex-smoker, current smoker, or non-smoker); hypertension (recorded hypertension or no hypertension). HbA1c = glycated haemoglobin. T2DM = type 2 diabetes mellitus.*

## Discussion

### Summary

A large nationally representative UK primary care database was used to assess the association between patient characteristics and orlistat prescribing, as well as orlistat’s associations with weight loss in patients with T2DM and prediabetes. The findings indicated that UK NICE guidelines for orlistat prescribing — that is, to adults with T2DM and a BMI ≥28 kg/m^2^ — were being followed, but that prescribing rates were low. Only <1% patients received orlistat and 42% of those received only one prescription, with no further prescription beyond 12 weeks; this indicated that they received little treatment. The prescribing of orlistat appeared to be higher in younger patients, females, those with lower HbA1c levels, those coprescribed metformin, those who had hypertension, and current smokers. Orlistat was prescribed more in areas of greater deprivation than in the most-affluent areas; this may reflect higher levels of severe obesity, consistent with findings from previous studies.^27^^,^^28^

The findings showed that continuing with orlistat was associated with ≥5% weight loss and greater reductions in systolic BP, and had a lower impact on HbA1c levels. Stopping early was associated with less reduction in HbA1c. Higher HbA1c levels were positively associated with stopping early.

### Strengths and limitations

To the authors’ knowledge, this is the largest study that has examined the real-world associations of orlistat with patient characteristics and weight loss in T2DM/prediabetes, and used a large, nationally representative primary care database in the UK. The current study does, however, have some limitations. Most importantly, the number of patients prescribed orlistat was limited, which can indicate difficulty with generalisation. Patient characteristics associated with orlistat prescribing were also examined only within T2DM, as numbers for those with prediabetes were very low; however, the groups were combined for the second objective, as orlistat was likely to have a similar effect in T2DM and prediabetes. It should be noted that some patients may have started orlistat as an over-the-counter medication — these are unrecorded in primary care electronic health records (EHRs), thereby underestimating orlistat’s incidence. Orlistat can be bought over the counter at a lower dose of 60 mg in the UK; even if offered it by GPs, patients may have refused it. Some confounding factors — for example, diet and exercise — are not captured in the CPRD, which can give rise to residual confounding. The reasons for stopping orlistat early are recorded as free text in EHRs (unavailable for research), and diet, exercise, or severity of T2DM are not well recorded in EHRs.

In order to explore only orlistat’s association with weight loss, the authors excluded patients on second-line antidiabetic medications/insulin, as these may also lead to weight loss. Metformin was only included as it is the first-line treatment in the UK and the antidiabetic medication that is most frequently prescribed.^29^ Weight data were only available for a subset of patients, which could cause bias if reasons for recording weight varied between orlistat users and non-users. Unexplained or unintentional weight loss can also be a symptom of new-onset diabetes — caused due to osmotic diuresis under high blood–glucose conditions^30^ — and this could partially explain some of the weight loss observed for some patients in all groups.

### Comparison with existing literature

The findings that UK NICE guidelines for orlistat prescribing were being followed is consistent with results of a previous UK study.^26^ Prescription rates were, however, low and the findings did not elucidate the reasons for this; it may be that the prescribing recommendations for orlistat are not being followed, an issue previously highlighted by Linné *et al*.^31^

Smokers and those with hypertension were more likely to be prescribed orlistat,^32^^–^^34^ possibly for cardiovascular disease risk reduction in T2DM.

This real-world study confirmed the weight-loss results of RCTs that underpinned the use of orlistat in T2DM and prediabetes; the levels of weight loss matched findings from RCTs in T2DM^35^^,^^36^ and a meta-analysis.^37^ Similar to the findings presented here, weight changes in a UK general population cohort using CPRD data showed an average of 2.2 kg reduction with orlistat in 3 years.^26^ A weight reduction of ≥5% has previously been considered clinically meaningful.^38^

Glycaemic parameters and systolic BP can improve with just ≥2.5% weight loss.^39^ Furthermore, even 1–2 kg of weight loss can be beneficial — for example, one study suggested that for each 1 kg of weight loss, T2DM risk decreases by 16%.^40^ However, weight-loss rates may be slower in T2DM so, as highlighted by NICE,^13^ less-strict goals may be considered. Weight-loss benefits may last for ≥5 years and, even if regained, improvements to blood-glucose levels remain.^41^

### Implications for research and practice

Low prescribing rates may be because individuals are prioritising lifestyle and dietary changes, and could imply a less-significant impact on health care; however, orlistat may not be prescribed for long in those with higher HbA1c values if it proves ineffective at reducing weight/HbA1c levels, as reducing high blood–glucose levels is a key priority in the treatment of T2DM. The low use of orlistat and early stopping could also be because GPs consider it ineffective or because patients are declining it, even if it is offered. There is also a possibility that GPs’ decisions may be influenced by the fact that orlistat’s side-effects can be intolerable and there may be adverse effects to its use. Orlistat is contraindicated in cholestasis/chronic malabsorption syndrome and it is advised that caution be observed in those with chronic kidney disease;^14^^,^^17^ however, the European Medicines Agency reviewed orlistat’s safety concerns in 2012 and concluded that its benefits outweigh the risks.^42^ There has also been a surge in second-line antidiabetic medication prescribing — for example, glucagon-like peptide-1 receptor agonists and sodium-glucose cotransporter-2 inhibitors — in recent times;^25^^,^^43^ these may have reduced the need for simultaneous orlistat prescribing alongside metformin in T2DM, due to their associations with greater weight loss.^43^ Further research on reasons for low prescription rates would be beneficial.

Continued use of orlistat was associated with targeted reductions in weight. Reductions in systolic BP may have important clinical implications in reducing major cardiovascular risks. Although other approaches — for example, diet and exercise — need to be considered first, lifestyle modification and weight loss/management, especially in T2DM/prediabetes, can be challenging for many. Although it needs to be seen whether weight loss with orlistat is maintained over longer follow-up periods, the medication could have an important role in T2DM/prediabetes to help people start losing weight if adherence to a change in lifestyle factors proves difficult. Patient comorbidities need to be considered, as well as orlistat’s potential side-effects, which may prove a barrier to the continued use of orlistat that may be needed for it to be effective.

Weight loss appeared to be sustainable in patients who continued treatment with orlistat, but there seem to be barriers to continuing its use and it may not be effective for everyone. Ideally, orlistat should be prescribed only after diet changes and exercise have been advised, and evaluated, for weight loss. Future studies need to examine the impact of orlistat alongside these lifestyle interventions, as well as its longer-term impact on weight loss. Larger studies are also needed as the power of this study was reduced by there only being a small number of patients prescribed orlistat, who were not on second-line medications and had recorded weight over time.

## Data

Data were obtained from the CPRD. CPRD data governance does not allow the authors to distribute patient data to other parties but researchers may apply for data access at: https://www.cprd.com.
